# Development of a machine learning-based model to predict major adverse events after surgery for type A aortic dissection complicated by malnutrition

**DOI:** 10.3389/fnut.2024.1428532

**Published:** 2024-07-04

**Authors:** Lin-feng Xie, Xin-fan Lin, Yu-ling Xie, Qing-song Wu, Zhi-huang Qiu, Quan Lan, Liang-wan Chen

**Affiliations:** ^1^Department of Cardiovascular Surgery, Fujian Medical University Union Hospital, Fuzhou, Fujian, China; ^2^Key Laboratory of Cardio-Thoracic Surgery (Fujian Medical University), Fujian Province University, Fuzhou, Fujian, China; ^3^Fujian Provincial Center for Cardiovascular Medicine, Fuzhou, Fujian, China; ^4^Department of Neurology, First Affiliated Hospital of Xiamen University, Xiamen, Fujian, China

**Keywords:** type A aortic dissection, machine learning, malnutrition, predictive model, major adverse events

## Abstract

**Objective:**

This study aims to develop a predictive model for the risk of major adverse events (MAEs) in type A aortic dissection (AAAD) patients with malnutrition after surgery, utilizing machine learning (ML) algorithms.

**Methods:**

We retrospectively collected clinical data from AAAD patients with malnutrition who underwent surgical treatment at our center. Through least absolute shrinkage and selection operator (LASSO) regression analysis, we screened for preoperative and intraoperative characteristic variables. Based on the random forest (RF) algorithm, we constructed a ML predictive model, and further evaluated and interpreted this model.

**Results:**

Through LASSO regression analysis and univariate analysis, we ultimately selected seven feature variables for modeling. After comparing six different ML models, we confirmed that the RF model demonstrated the best predictive performance in this dataset. Subsequently, we constructed a model using the RF algorithm to predict the risk of postoperative MAEs in AAAD patients with malnutrition. The test set results indicated that this model has excellent predictive efficacy and clinical applicability. Finally, we employed the Shapley additive explanations (SHAP) method to further interpret the predictions of this model.

**Conclusion:**

We have successfully constructed a risk prediction model for postoperative MAEs in AAAD patients with malnutrition using the RF algorithm, and we have interpreted the model through the SHAP method. This model aids clinicians in early identification of high-risk patients for MAEs, thereby potentially mitigating adverse clinical outcomes associated with malnutrition.

## Introduction

Aortic dissection (AD) is an extremely dangerous cardiovascular emergency, especially type A aortic dissection (AAAD). Without prompt surgical intervention, its mortality rate increases by approximately 1%–2% per hour, with a mortality rate as high as 50% within 48 hours (1–3). Despite significant advancements in surgical techniques and life support technologies such as cardiopulmonary bypass in recent years, the prognosis of AAAD remains relatively poor compared to other cardiovascular diseases (4). Therefore, effectively assessing and predicting the risk of postoperative adverse clinical events is crucial before making diagnostic and therapeutic decisions for AAAD patients.

Malnutrition is a common comorbidity upon admission and also a significant adverse prognostic factor for cardiovascular diseases (5, 6). Research by Shirley suggests a close association between malnutrition and mortality in patients with atrial fibrillation (7). Similarly, studies by Al-Kassou et al. (8) indicate that malnutrition increases the mortality rate following aortic valve surgery. However, there is currently limited research on the relationship between malnutrition and the prognosis of AAAD. Additionally, there is also a lack of a model that can effectively predict the clinical outcomes of AAAD patients with concurrent malnutrition following surgical treatment.

With the widespread adoption and popularization of artificial intelligence technology, its application in the field of biomedicine is rapidly advancing. Machine learning (ML), as a specialized form of artificial intelligence, has been widely utilized in the diagnosis and treatment of various diseases and their prognosis (9, 10). Compared to traditional logistic regression prediction models, ML models demonstrate greater flexibility and accuracy in handling complex, non-linear relationships within data (11–13). This study aims to construct a prognosis model based on ML algorithms to predict the risk of major adverse events (MAEs) in-hospital for AAAD patients with concurrent malnutrition following surgical treatment.

## Materials and methods

### Study population

This study retrospectively analyzed the clinical data of AAAD patients over 18 years old who underwent surgical treatment consecutively at our center from January 2018 to January 2022. The exclusion criteria were as follows: (1) chronic aortic dissection, (2) patients with preoperative comorbidities such as malignant tumors, hematologic disease, systemic inflammatory diseases, (3) history of previous thoracotomy, (4) significant lack of medical history data. This study was approved by the Ethics Committee of Fujian Medical University Union Hospital and complied with the Helsinki declaration. Informed consent was waived due to the retrospective nature of the study.

### Definition and endpoint

The nutritional risk index (NRI) is a commonly used clinical nutritional assessment tool. In this study, the patients’ nutritional status upon admission was assessed using the NRI (14). The calculation of the NRI follows the formula proposed by Buzby: 1.519 * serum albumin (g/l) + 41.7 * (current body weight [kg]/usual body weight [kg]). Usual body weight is replaced by ideal body weight, with the formula for calculating ideal body weight in male as follows: height (cm) − 100 − ([height (cm) − 150]/4), and for female, the formula is: height (cm) − 100 − ([height (cm) − 150]/2.5). When the current body weight exceeds the ideal body weight, we set the ratio of current body weight to ideal body weight as 1 (14–16). Taking into account the application of the NRI in other cardiovascular disease studies, we defined patients with NRI < 97.5 upon admission as having malnutrition.

The main endpoint of this study is the occurrence of MAEs during hospitalization. According to the consensus statement from the international study group for aortic arch surgery on the grading criteria for complications after aortic arch surgery (17), MAEs include: (1) cardiovascular complications (myocardial infarction, malignant arrhythmias, and heart failure requiring support with intra-aortic balloon pump), (2) respiratory complications [acute lung injury, acute respiratory distress syndrome, prolonged mechanical ventilation, reintubation, tracheostomy, or respiratory failure requiring extracorporeal membrane oxygenation (ECMO) therapy], (3) new-onset acute kidney injury [serum creatinine level increased more than three times baseline or renal failure requiring continuous renal replacement therapy (CRRT)], (4) gastrointestinal bleeding, (5) wound complications requiring reoperation for hemostasis or further surgical intervention, (6) postoperative death.

### Data collection

Clinical data of AAAD patients were collected through the hospital’s electronic medical record system, including demographic information such as gender, age, and body mass index (BMI), as well as past medical history including hypertension, diabetes, and coronary artery disease (CAD). Preoperative comorbidities such as chronic kidney disease (CKD), aortic valve regurgitation, and pericardial effusion were also documented. Preoperative laboratory test results including leukocyte, neutrophil, monocyte, lymphocyte, etc. were recorded. Intraoperative details such as operation time, cardiopulmonary bypass time, and aortic cross-clamp time were documented. Postoperative clinical outcomes included ICU stay time, mechanical ventilation time, 48 hours thoracic drainage, and postoperative complications.

### Model training and performance evaluation

All AAAD patients with malnutrition were randomly divided into training and test datasets at a ratio of 8:2. The training dataset was used for modeling purposes, while the test dataset was utilized for model evaluation. In the training set, LASSO regression analysis was used to select feature variables related to MAEs from preoperative and intraoperative variables. Models were then constructed based on algorithms such as eXtreme Gradient Boosting (XGBoost), Logistic Regression (LR), Random Forest (RF), Multilayer Perceptron (MLP), Support Vector Machine (SVM), and K-Nearest Neighbors (KNN). To prevent overfitting, we employed a 10-fold resampling validation by further divided the training set into 10 subsets. During each iteration, nine of the subsets are used to train the model, while the remaining one subset is used for validation. Subsequently, we performed grid search for hyperparameter tuning to select the optimal model (Supplementary Table S1). In the final evaluation on the training set data, the model performance was assessed from three dimensions: model discrimination, predictive accuracy, and clinical applicability. Model discrimination was quantitatively evaluated through metrics such as the area under the ROC curve (AUC), accuracy, sensitivity, specificity, positive predictive value, negative predictive value, and F1 score. Predictive accuracy was assessed by comparing the deviation between predicted probabilities and actual probabilities (Supplementary Tables S2, S3). Clinical applicability was judged through the decision curve analysis (DCA).

After determining the optimal model, we further validated its performance using the test set data. Additionally, we constructed calibration curves, DCA curves, and model learning curves for the optimal model. Finally, we utilized the Shapley additive explanations (SHAP) method to further explain the clinical significance of the model.

### Statistical analysis

All data were analyzed using SPSS 24.0, R 4.2.1, and Python 3.7. Continuous data were expressed as mean ± standard deviation (SD) or median (interquartile range), and analyzed using Student’s *t*-test or Mann–Whitney-*U* test. Categorical data were presented as frequency or percentage (%), and analyzed using chi-square test or Fisher’s exact test. Initially, all AAAD patients with combined malnutrition were randomly divided into training and test sets at an 8:2 ratio. In the training set, clinical feature variables were selected through LASSO regression analysis, and models were built using six ML algorithms. The best model was selected and its performance was validated. Finally, the SHAP method was used to explain the model. A two-tailed *p* < 0.05 was considered statistically significant.

## Results

### Patient characteristics

In this study, a total of 708 patients with AAAD were included, among which 308 patients (43.5%) presented with malnutrition upon hospital admission. We compared the postoperative clinical outcomes of patients with normal nutrition and malnutrition (Table 1), and the results showed that compared to the normal nutrition group, the malnutrition group had increased thoracic drainage volume within 48 hours postoperatively, prolonged mechanical ventilation time, ICU stay, and postoperative hospital stay. Additionally, the proportion of patients requiring CRRT, ECMO therapy, as well as the occurrence of AKI, permanent neurological dysfunction (PND), and low cardiac output syndrome (LCOS) increased. The incidence of MAEs during hospitalization (28.25% vs. 18.00%) and in-hospital mortality rate (12.99% vs. 5.28%) were significantly higher (*p* < 0.05).

**Table 1:** Comparison of postoperative outcomes between patients with normal nutrition and malnutrition.

Valuables	Normal nutrition group (*n* = 400)	Malnutrition group (*n* = 308)	*P-*value
Hospital stay (day)	18.60[16.00,22.00]	19.00[17.00,22.00]	**0.015**
ICU stay (day)	5.00[4.00,7.00]	6.00[5.00,8.00]	**<0.001**
Thoracic drainage (mL/48 h)	780.00[570.00,1050.00]	800.00[610.00,1230.00]	**0.031**
Mechanical ventilation time (h)	54.00[29.00,73.00]	63.00[37.00,81.00]	**0.002**
Need CRRT, (*n*, %)	44(11.00)	64(20.78)	**<0.001**
Need ECMO, (*n*, %)	2(0.50)	8(2.60)	**0.019**
AKI (*n*, %)	64(16.00)	81(26.30)	**<0.001**
PND (*n*, %)	16(4.00)	29(9.42)	**0.003**
LCOS (*n*, %)	5(1.25)	19(6.17)	**<0.001**
GB (*n*, %)	17(4.25)	14(4.55)	0.849
Sepsis (*n*, %)	5(1.25)	2(0.65)	0.423
Secondary intubation (*n*, %)	29(7.25)	24(7.79)	0.786
Tracheotomy (*n*, %)	18(4.50)	21(6.82)	0.180
Secondary thoracotomy (*n*, %)	1(0.25)	3(0.97)	0.203
In-hospital mortality (%)	21(5.25)	40(12.99)	**<0.001**
MAEs (*n*, %)	72(18.00)	87(28.25)	**0.001**

CRRT, continuous renal replacement therapy; ECMO, extracorporeal membrane oxygenation; AKI, acute kidney injury; PND, permanent neurological dysfunction; LCOS, low cardiac output syndrome; GB, gastrointestinal bleeding; MAEs, major adverse events. Bold indicates statistically significant differences.

**Figure 1:**
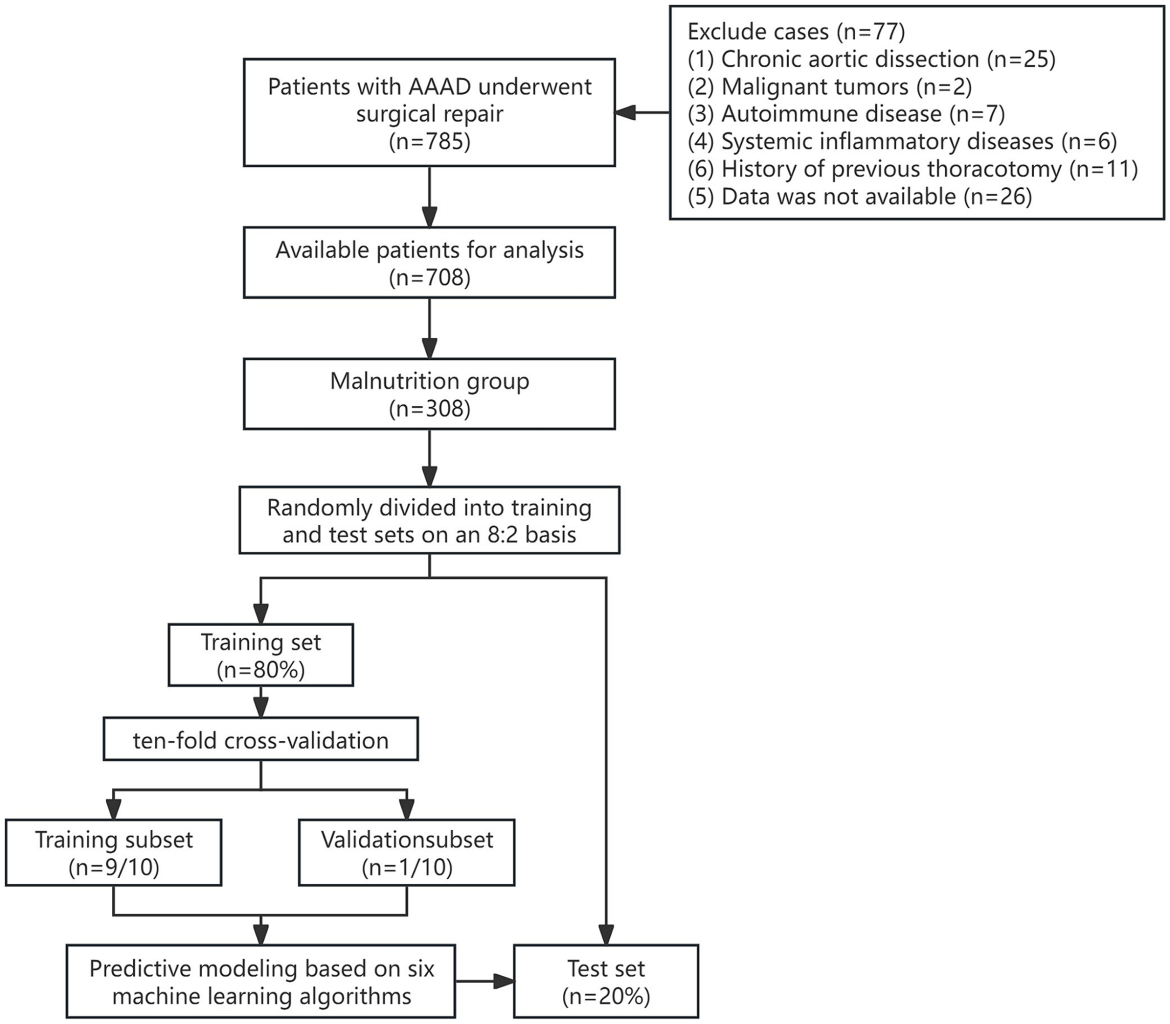
Model construction methodology and study flowchart.

Among AAAD patients with malnutrition, 246 patients (80%) were randomly assigned to the training set, while 62 patients (20%) were assigned to the test set. The workflow diagram of the study is shown in Figure 1. There were no significant differences in demographic data, preoperative comorbidities, or intraoperative conditions between the two groups (Table 2).

**Table 2:** Baseline characteristics of enrolled patients.

Valuables	Training set (*n* = 246)	Test set (*n* = 62)	*P-*value
Demographical data			
Age, (years)	57.00[46.00,65.00]	54.00[48.00,63.00]	0.943
Gender, (male)	149(60.57)	38(61.29)	0.917
Body mass index, (kg/m^2^)	23.91 ± 4.12	23.66 ± 3.71	0.666
NRI	89.40[84.09,94.26]	88.64[84.99,95.62]	0.388
Risk factors and comorbidities			
Smoking, *n* (%)	110(44.72)	24(38.71)	0.394
Alcohol, *n* (%)	38(15.45)	9(14.52)	0.855
Hypertension, *n* (%)	158(64.23)	43(69.35)	0.449
Diabetes, *n* (%)	9(3.66)	1(1.61)	0.417
Previous CAD, *n* (%)	1(0.41)	0	1.000
Previous CVD, *n* (%)	9(3.66)	2(3.23)	0.870
Previous CKD, *n* (%)	5(2.03)	0	0.569
Marfan Syndrome, *n* (%)	8(3.25)	1(1.61)	0.493
Pericardial effusion (Medium or above), *n* (%)	24(9.76)	5(8.06)	0.684
Aortic valve regurgitation (Medium or above), *n* (%)	52(21.14)	13(20.97)	0.977
Preoperative laboratory results			
Leukocyte, (×10ˆ9/L)	12.15[9.77,15.36]	11.55[9.26,15.49]	0.432
Neutrophil, (×10ˆ9/L)	10.44[7.84,13.42]	9.97[7.39,13.22]	0.418
Monocyte, (×10ˆ9/L)	0.66[0.46,0.96]	0.69[0.35,0.92]	0.432
Lymphocyte, (×10ˆ9/L)	0.83[0.54,1.15]	0.84[0.53,1.21]	0.850
HB, (g/L)	130.00[117.00,142.00]	127.00[118.00,141.00]	0.682
PLT, (×10ˆ9/L)	175.00[141.00,214.00]	176.00[140.00,206.00]	0.928
ALB, (g/L)	32.10[28.90,35.00]	31.80[28.80,35.50]	0.743
Creatinine, (μmol/L)	85.00[66.00,117.00]	80.00[63.70,106.00]	0.448
BUN, (mmol/L)	6.40[5.21,8.50]	5.93[4.70,7.29]	0.108
D-dimer, (μg/mL)	13.02[6.70,20.00]	8.46[4.75,18.86]	0.129
BNP, (pg/mL)	340.00[150.00,844.00]	258.00[113.00,601.00]	0.255
Troponin-I, (μg/L)	0.01[0.00,0.09]	0.01[0.00,0.05]	0.389
CRP (mg/L)	10.40[4.58,38.23]	11.52[7.05,43.94]	0.196
Intraoperative conditions			
Ascending aorta replacement, *n* (%)	244(99.19)	62(100.00)	1.000
Root surgery			0.597
Untreated	58(23.58)	17(27.42)	
Reconstruction of sinus of valsalva	117(47.56)	31(50.00)	
Bentall	62(25.20)	14(22.58)	
Wheat	7(2.85)	0	
David	2(0.81)	0	
CABG, (*n*, %)	9(3.66)	3(4.84)	0.668
Mitral surgery, (*n*, %)	1(0.41)	1(1.61)	0.291
TVP, (*n*, %)	6(2.44)	0	0.467
Operation time, (min)	256.00[225.00,290.00]	260.00[225.00,320.00]	0.343
CPB time, (min)	155.00[137.00,184.00]	169.00[138.00,192.00]	0.255
ACC time, (min)	101.00[86.00,130.00]	102.00[90.00,134.00]	0.413
DHCA time, (min)	12.00[12.00,13.00]	13.00[12.00,13.00]	0.998
Plasma transfusion volume, (mL)	200.00[200.00,400.00]	200.00[200.00,350.00]	0.538
RBC transfusion volume, (U)	4.00[0.00,4.00]	3.00[0.00,4.00]	0.099
Platelet transfusion volume, (U)	1.00[0.80,10.00]	4.00[1.00,8.00]	0.660

NRI, nutritional risk index; CAD, coronary artery disease; CVD, cerebrovascular disease; CKD, chronic kidney disease; HB, hemoglobin; PLT, platelet; ALB, albumin; BUN, blood urea nitrogen; BNP, B-type natriuretic peptide; CRP, C-reactive protein; CABG, coronary artery bypass grafting; TVP, tricuspid valvuloplasty; CPB, cardiopulmonary bypass; ACC, aortic cross clamp; DHCA, deep hypothermic circulatory arrest; RBC, red blood cell.

### Feature variable selection

**Figure 2:**
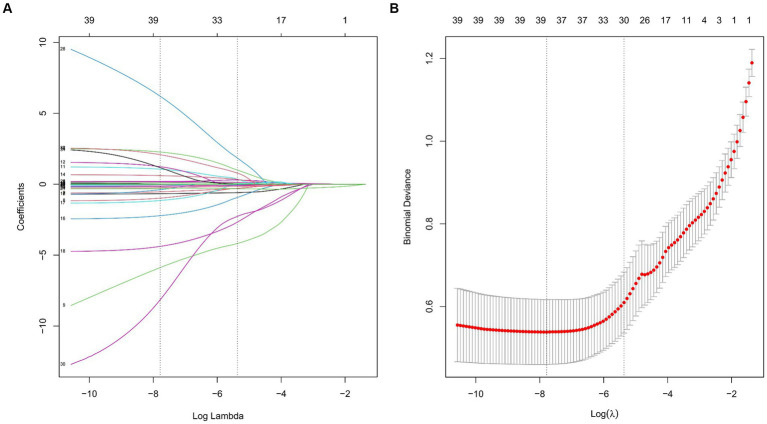
Feature variable selection based on the LASSO regression analysis. **(A)** Plot of the LASSO coefficient profiles. **(B)** Tuning parameter selection cross-validation error curve. (LASSO, least absolute shrinkage and selection operator).

Through LASSO regression analysis, preliminary screening of feature variables related to postoperative MAEs was conducted. The result indicated a minimum mean square error lambda value of 0.004, including 35 preoperative and intraoperative feature variables (Figure 2). Subsequently, in both the training and test sets, univariate analysis was further performed to compare preoperative and intraoperative conditions between the MAEs group and the non-MAEs group (Table 3). Finally, seven feature variables, including preoperative NRI, preoperative hypertension, preoperative leukocyte, preoperative lymphocyte, preoperative albumin (ALB), preoperative D-dimer, and preoperative C-reactive protein (CRP), were determined to construct the clinical feature variables for building the ML model.

**Table 3:** Comparison of preoperative and intraoperative conditions between the MAEs group and the non-MAEs group in different datasets.

Valuables	Training set (*n* = 246)			Test set (*n* = 62)		
	Non-MAEs group (*n* = 179)	MAEs group (*n* = 67)	*P-*value	Non-MAEs group (*n* = 42)	MAEs group (*n* = 20)	*P-*value
Demographical data						
Age, (years)	57.00[47.00,65.00]	56.00[45.00,64.00]	0.442	55.29 ± 11.92	57.60 ± 10.63	0.470
Gender, (male)	105(58.66)	44(65.67)	0.316	25(59.52)	13(65.00)	0.679
Body mass index, (kg/m^2^)	23.79 ± 3.99	24.21 ± 4.42	0.480	23.24[20.90,25.62]	23.59[21.37,26.26]	0.970
NRI	91.66[86.97,94.87]	84.03[78.31,86.66]	**<0.001**	93.50[87.73,96.07]	86.36[83.47,88.03]	**0.011**
Risk factors and comorbidities						
Smoking, *n* (%)	81(45.25)	29(43.28)	0.782	14(33.33)	10(50.00)	0.208
Alcohol, *n* (%)	26(14.53)	12(17.91)	0.513	4(9.52)	5(25.00)	0.106
Hypertension, *n* (%)	123(68.72)	35(52.24)	**0.016**	28(66.67)	15(75.00)	0.506
Diabetes, *n* (%)	7(3.91)	2(2.99)	0.731	0(0.00)	1(5.00)	0.323
Previous CAD, *n* (%)	1(0.56)	0	1.000	0	0	1.000
Previous CVD, *n* (%)	6(3.35)	3(4.48)	0.675	2(4.76)	0	1.000
Previous CKD, *n* (%)	3(1.68)	2(2.99)	0.517	0	0	1.000
Marfan Syndrome, *n* (%)	6(3.35)	2(2.99)	0.885	1(2.38)	0	1.000
Pericardial effusion (medium or above), *n* (%)	18(10.06)	6(8.96)	0.796	4(9.52)	1(5.00)	0.541
Aortic valve regurgitation (medium or above), *n* (%)	40(22.35)	12(17.91)	0.448	8(19.05)	5(25.00)	0.590
Preoperative laboratory results						
Leukocyte, (×10ˆ9/L)	11.66[9.39,14.55]	13.99[11.23,17.58]	**<0.001**	11.49 ± 3.25	13.97 ± 4.39	**0.017**
Neutrophil, (×10ˆ9/L)	10.17[7.64,13.23]	10.93[8.18,13.92]	0.130	9.71 ± 3.30	11.50 ± 4.20	0.078
Monocyte, (×10ˆ9/L)	0.64[0.45,0.90]	0.74[0.48,1.09]	0.068	0.75[0.38,0.97]	0.43[0.33,0.72]	0.124
Lymphocyte, (×10ˆ9/L)	0.85[0.60,1.15]	0.69[0.43,1.16]	**0.026**	1.06 ± 0.50	0.62 ± 0.37	**0.001**
HB, (g/L)	130.00[117.00,144.00]	129.00[115.00,137.00]	0.111	128.98 ± 17.01	126.15 ± 21.26	0.582
PLT, (×10ˆ9/L)	178.00[140.00,217.00]	170.00[142.00,209.00]	0.859	166.00[140.00,225.00]	186.00[138.00,202.00]	0.695
ALB, (g/L)	34.00[31.10,35.80]	28.30[26.10,30.20]	**<0.001**	35.00[30.40,36.10]	29.90[27.50,30.50]	**<0.001**
Creatinine, (μmol/L)	85.00[66.00,113.00]	79.00[67.00,147.00]	0.780	71.00[52.80,104.00]	96.00[80.00,119.00]	**0.039**
BUN, (mmol/L)	6.30[5.20,8.00]	6.88[5.30,9.80]	0.173	5.80[4.70,7.30]	6.40[5.30,6.90]	0.583
D-dimer, (μg/mL)	11.35[4.34,19.90]	17.30[9.85,20.00]	**<0.001**	5.97[3.71,12.21]	19.60[10.00,20.00]	**<0.001**
BNP, (pg/mL)	288.00[135.00,834.00]	439.00[175.00,1047.00]	0.078	199.00[110.00,601.00]	306.00[189.00,527.00]	0.470
Troponin-I, (μg/L)	0.01[0.00,0.09]	0.02[0.01,0.13]	0.071	0.01[0.00,0.03]	0.02[0.01,0.17]	0.074
CRP (mg/L)	10.29[3.12,29.40]	17.40[6.80,76.30]	**0.002**	14.15[6.47,38.10]	11.52[7.80,48.80]	0.792
Intraoperative conditions						
Ascending aorta replacement, *n* (%)	177(98.88)	67(100.00)	1.000	42(100.00)	20(100.00)	1.000
Root surgery			0.713			0.933
Untreated	45(25.14)	13(19.40)		12(28.57)	5(25.00)	
Reconstruction of sinus of valsalva	84(46.93)	33(49.25)		21(50.00)	10(50.00)	
Bentall	45(25.14)	17(25.37)		9(21.43)	5(25.00)	
Wheat	4(2.23)	3(4.48)		0	0	
David	1(0.56)	1(1.49)		0	0	
CABG, (*n*, %)	9(5.03)	0	0.119	3(7.14)	0	0.545
Mitral surgery, (*n*, %)	0	1(1.49)	0.272	1(2.38)	0	1.000
TVP, (*n*, %)	4(2.23)	2(2.99)	0.734	0	0	1.000
Operation time, (min)	257.00[223.00,290.00]	253.00[233.00,291.00]	0.867	260.00[220.00,325.00]	271.00[240.00,320.00]	0.451
CPB time, (min)	155.00[136.00,182.00]	162.00[140.00,187.00]	0.169	156.00[135.00,190.00]	174.00[158.00,206.00]	0.089
ACC time, (min)	100.00[85.00,127.00]	104.00[86.00,140.00]	0.402	95.00[88.00,134.00]	116.00[102.00,134.00]	0.195
DHCA time, (min)	12.00[12.00,13.00]	13.00[12.00,13.00]	0.358	13.00[12.00,13.00]	12.00[12.00,13.00]	0.346
Plasma transfusion volume, (mL)	250.00[200.00,400.00]	200.00[200.00,400.00]	0.634	200.00[0.00,349.50]	250.00[200.00,400.00]	0.379
RBC transfusion volume, (U)	4.00[0.00,4.00]	4.00[0.00,4.00]	0.384	4.00[0.00,4.00]	3.00[0.00,4.00]	0.987

NRI, nutritional risk index; CAD, coronary artery disease; CVD, cerebrovascular disease; CKD, chronic kidney disease; HB, hemoglobin; PLT, platelet; ALB, albumin; BUN, blood urea nitrogen; BNP, B-type natriuretic peptide; CRP, C-reactive protein; CABG, coronary artery bypass grafting; TVP, tricuspid valvuloplasty; CPB, cardiopulmonary bypass; ACC, aortic cross clamp; DHCA, deep hypothermic circulatory arrest; RBC, red blood cell.

### Comparative analysis of multiple models and validation of the optimal model

**Figure 3:**
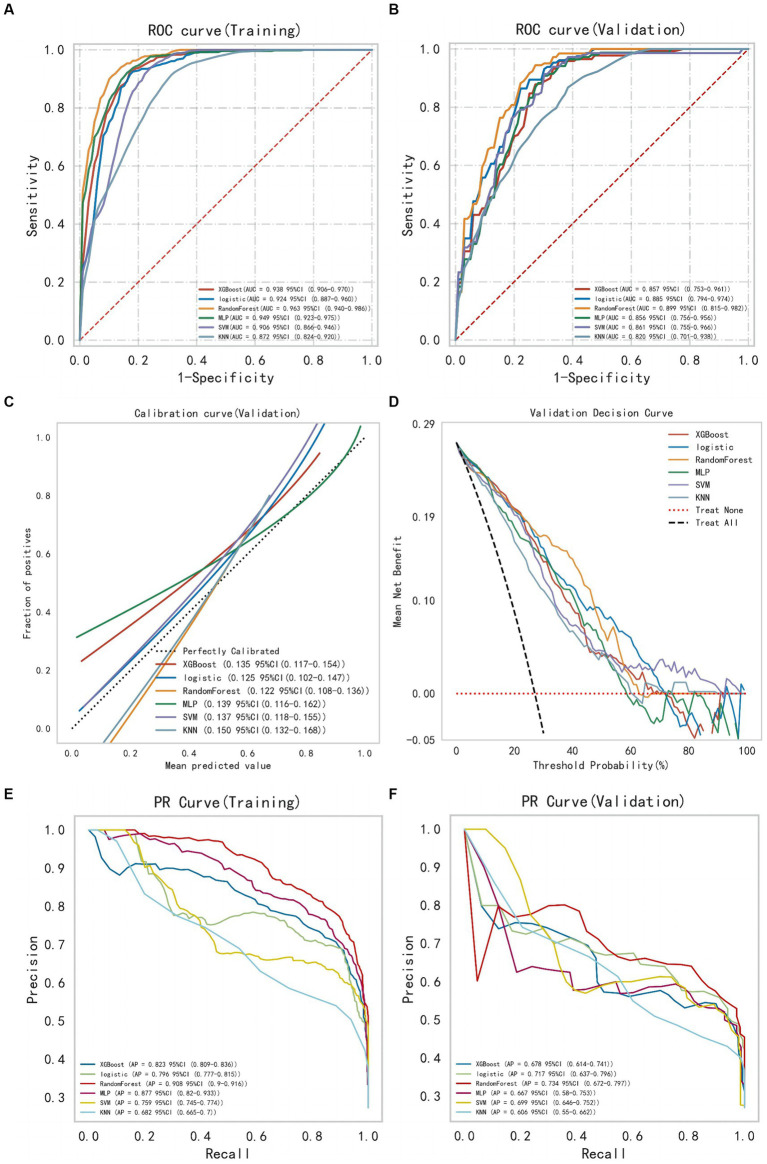
The comprehensive analysis of six machine learning models. **(A)** The ROC curve and AUC of the training set. **(B)** The ROC curve and AUC of the validation set. **(C)** The calibration curve plot of six models. **(D)** The DCA curve of the validation set. **(E)** The PR curve of the training set. **(F)** The PR curve of the validation set (ROC, receiver operating characteristic; AUC, area under the curve; DCA, decision curve analysis; PR, precision recall).

After selecting the required feature variables for modeling, the XGBoost, LR, RF, MLP, SVM, and KNN algorithms were employed to analyze the training set data, and the discriminative performance of the models was evaluated through AUC. The AUC of all models was validated through 10-fold resampling-validation. The final results show that among all models, the RF model demonstrates the best performance both on the training and validation sets (Figure 3). The AUC for RF model on the training set is 0.963 [95% Confidence Interval (CI): 0.940–0.986], and on the validation set, it is 0.899 (95% CI: 0.815–0.982). The Brier score (95% CI) for the RF model in the calibration curve is 0.122 (95% CI: 0.108–0.136), indicating good calibration. The DCA also illustrates its favorable clinical utility. Moreover, the precision-recall curve results indicate that the RF model has the highest average precision values on both datasets. Therefore, we conclude that the RF model is the optimal model choice for this dataset.

**Figure 4:**
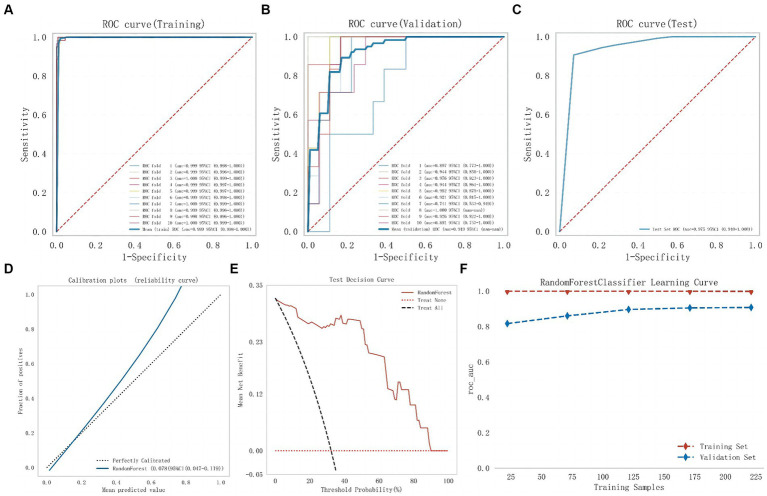
The comprehensive analysis of Random Forest model. **(A)** The ROC curve and AUC of the training set. **(B)** The ROC curve and AUC of the validation set. **(C)** The ROC curve and AUC of the test set. **(D)** The calibration curve plot of the Random Forest model. **(E)** The DCA curve of the Random Forest model. **(F)** Random Forest model learning curve (ROC, receiver operating characteristic; AUC, area under the curve; DCA, decision curve analysis).

Subsequently, modeling and 10-fold cross-validation were conducted on the test dataset using the RF algorithm (Figure 4). The results indicate that the average AUC on the training set is 0.999, and on the validation set, it is 0.919. Ultimately, the RF model achieves an average AUC of 0.975 on the test set with an accuracy of 88.71%. The calibration curve results reveal a Brier Score of 0.078 (95%CI: 0.047–0.119) for the RF model, indicating its good predictive accuracy. Furthermore, the DCA results also demonstrate its favorable clinical utility. The results of the model learning curve indicate a good fit of the RF model on both the training and validation sets.

### Model explanation and clinical significance analysis

To further elucidate the clinical significance of this model, we explained the prediction process and results of the RF model through the SHAP method. Based on the concept of the Shapley value in cooperative game theory, we quantify the contribution of each feature to the model’s output and calculate the SHAp value for each feature variable to assess the impact of each feature on a single prediction made by the model.

**Figure 5:**
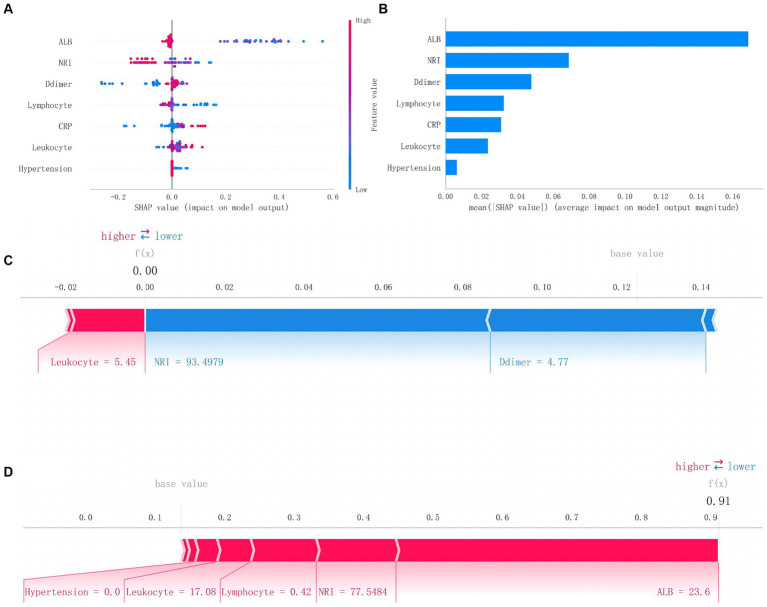
SHAP analysis of Random Forest model. **(A)** The scatter plot of feature distributions using the SHAP analysis. **(B)** Ranking feature importance based on the absolute mean values of SHA*p* values. **(C)** Force plot for patients in the testing set with MAEs. **(D)** Force plot for patients in the testing set without MAEs (SHAP, Shapley additive explanations; MAEs, major adverse events).

Figure 5 presents the SHAP summary plot and the ranking of feature variables based on their impact on MAEs. Additionally, we further elucidate the model through two different samples from the test dataset: one where the model predicts no postoperative MAEs, and indeed, no MAEs occur *f*(*x*) = 0, and another where the model predicts postoperative MAEs, and MAEs actually occur *f*(*x*) = 0.91.

## Discussion

Malnutrition, as a common comorbidity upon admission, increases the risk of complications during hospitalization and is a key factor affecting the prognosis of many diseases (18, 19). There are several scoring systems used clinically to assess the nutritional status of hospitalized patients, such as the controlling nutritional status score and the prognostic nutritional index (20, 21). Among them, the NRI serves as a simple and effective scoring indicator. It primarily evaluates patients’ nutritional status based on serum albumin, actual body weight, and ideal body weight. Initially developed as a risk scoring tool to assess the nutritional status of elderly hospitalized patients and the incidence of malnutrition-related complications and mortality, NRI has gained popularity in recent years due to its simplicity, universality, and strong prognostic value across different surgical patient populations (14). However, there is currently no research specifically applying the NRI in patients with AAAD. Therefore, we referred to the application of NRI in other cardiovascular diseases. Patients with an NRI < 97.5 upon admission were diagnosed as having malnutrition (22).

By comparing postoperative outcomes between the normal nutrition group and the malnutrition group of patients with AAAD, we found that patients who had malnutrition experienced significantly prolonged mechanical ventilation time, ICU stay, total hospital days, and increased incidence of serious complications. Their short-term postoperative prognosis was poorer. Therefore, there is an urgent need for a reliable and effective predictive model to stratify the early risk and assess the prognosis of AAAD patients with malnutrition, aiming to improve the adverse postoperative outcomes caused by malnutrition.

This study represents the first application of artificial intelligence in prognostic prediction for AAAD patients with malnutrition. We attempted to establish six ML models and evaluated their efficacy in terms of model discrimination, accuracy, and clinical applicability. Ultimately, we successfully developed a ML model capable of predicting the risk of MAEs following surgery in AAAD patients with malnutrition. Compared to traditional logistic regression models, ML has greater flexibility, generalization ability, and accuracy in predictive model construction. As one of the most common ML algorithms, the RF algorithm has the advantage of higher accuracy, stronger resistance to overfitting, ease of interpretation, and suitability for large-scale data. Moreover, RF demonstrates robustness against missing data and outliers and provides assessments of the importance of each feature variable, aiding in understanding their contributions to the model’s operation (23, 24).

To further interpret this predictive model, we utilized the SHAP method to generate feature density scatter plots and feature importance ranking plots based on SHAP values. We described the contributions of the included feature variables to postoperative MAEs and the actual predictive results in the test dataset, enhancing the interpretability of the model.

The feature importance ranking results indicate that preoperative ALB, NRI, and D-dimer are the top three feature variables in the RF model. ALB, as a crucial plasma protein, plays a vital role in maintaining normal plasma oncotic pressure and balancing fluid within and outside blood vessels. Additionally, it holds significant value in reflecting long-term nutritional status. Hutter et al. (25) have reported a strong correlation between preoperative ALB levels and postoperative complications in male surgical patients. The relationship between preoperative low ALB levels and postoperative mortality has also been widely recognized in general surgical patients (26). In contrast, in malnourished patients, low albumin tends to be more likely to lead to decreased immune function and organ insufficiency, which increases the risk of postoperative MAEs.

The NRI, as an indicator of nutritional risk, not only takes into account serum albumin levels but also considers the patient’s weight body changes. According to research by Jabbour et al. (27), when both serum albumin levels and weight body alteration occur simultaneously, their impact on postoperative outcomes surpasses that of age, which is known to be a strong predictor of prognosis. A lower NRI indicates a higher risk of malnutrition and, consequently, a greater risk of adverse postoperative outcomes. Therefore, the NRI is also identified as a crucial factor influencing prognosis.

D-dimer is commonly regarded as a reliable indicator of coagulation and fibrinolysis, given that the systemic inflammatory storm induced by AAAD persistently activates both endogenous coagulation and fibrinolysis. Consequently, D-dimer levels in the serum of AAAD patients are significantly elevated. Its prognostic value in different types of aortic dissection has been widely acknowledged (28). The elevation of D-dimer levels is closely associated with the extensive formation of false lumen in preoperative dissection, and high D-dimer levels often indicate a more severe condition and a higher risk of postoperative complications. Thus, it serves as an effective predictive variable for postoperative MAEs.

Other predictive model features, including leukocyte, lymphocytes, and CRP, signify the significant association between inflammatory responses and immune activation with the prognostic outcomes in malnourished patients. Nutrition influences all physiological processes, including those related to immune system development and function (29). Chronic inflammation present in malnourished individuals can weaken the organ’s resilience against disease stress (30). Thereby making immune-inflammatory blood cells and biomarkers relevant predictors for potential postoperative complications.

In conclusion, we have successfully developed a ML model based on the RF algorithm, which effectively predicts the risk of postoperative MAEs in AAAD patients with malnutrition. This model has demonstrated impressive predictive performance in both validation and testing sets. Through this model, clinicians can early identify high-risk patients with malnutrition among AAAD patients, facilitating risk stratification and decision-making. This, in turn, can help reduce the adverse clinical outcomes associated with malnutrition and improve patients’ short-term prognosis.

### Limitations

This study still has the following limitations. Firstly, being a single-center retrospective study, it inherently carries some biases. Secondly, the data for the training and test sets are from different periods of the same center, thus lacking external data from multiple centers to further validate the model’s efficacy and clinical utility. Therefore, further multicenter, large-sample randomized clinical trials are still needed to corroborate our research findings. Finally, due to the lack of unified diagnostic criteria for malnutrition, different nutritional scoring systems diagnose malnutrition at vastly different rates. Although this study selected the widely used NRI as the evaluation basis, further integration with other types of nutritional scoring systems is still necessary to improve the assessment of prognosis risk for AAAD surgical patients with malnutrition.

## Conclusion

This study, for the first time, successfully developed and validated a predictive model based on ML algorithms for predicting the risk of postoperative MAEs in AAAD patients with malnutrition. The model demonstrated excellent predictive performance and clinical applicability. It can provide clinicians with a reliable basis for assessing the postoperative outcomes of AAAD patients with malnutrition, facilitating early risk stratification and decision-making.

## Data availability statement

The original contributions presented in the study are included in the article/Supplementary material, further inquiries can be directed to the corresponding authors.

## Author contributions

L-fX: Conceptualization, Investigation, Software, Writing – original draft, Writing – review & editing. X-fL: Conceptualization, Investigation, Software, Writing – original draft, Writing – review & editing. Y-lX: Conceptualization, Investigation, Software, Writing – original draft, Writing – review & editing. Q-sW: Data curation, Software, Writing – original draft, Writing – review & editing. Z-hQ: Funding acquisition, Resources, Validation, Writing – original draft, Writing – review & editing. QL: Validation, Writing – original draft, Writing – review & editing. L-wC: Funding acquisition, Resources, Validation, Visualization, Writing – original draft, Writing – review & editing.
