# Supplementary material for: Development of a machine learning-based model to predict major adverse events after surgery for type A aortic dissection complicated by malnutrition
Source: Front Nutr. 2024 Jul 4;11:1428532. doi: 10.3389/fnut.2024.1428532 (PMC11254848; doi:10.3389/fnut.2024.1428532)
Supplement: Supplementary file 1 [file Table_1.DOCX]

| **Supplement Table 1**. Selection of parameter values in multi-model comparisons. | |
| --- | --- |
| Model category | Model parameters |
| XGBoost | **objective**: reg:logistic  **learning rate**: 0.3  **max depth**: 6  **min child weight**: 10  **reg lambda**: 1 |
| LR | **C**: 1  **max iter**: 100  **penalty**: l2  **tol**: 0.0001 |
| RF | **criterion**: gini  **max depth**: 2  **min impurity decreas**e: 0  **n estimators**: 100 |
| MLP | **activation**: logistic **hidden layer sizes**: (200,200) **max iter**: 200 |
| SVM | **C**: 1.0  **kernel**: poly  **tol**: 0.1 |
| KNN | **n neighbors**: 20  **weights**: uniform |
| **XGBoost**, eXtreme Gradient Boost; **LR**, Logistic Regress; **RF**, Random Forest;  **MLP**, Multilayer Perceptron; **SVM**, Support Vector Machines; **KNN**, K-Nearest Neighbor. | |
