# Supplementary material for: Development of a machine learning-based model to predict major adverse events after surgery for type A aortic dissection complicated by malnutrition
Source: Front Nutr. 2024 Jul 4;11:1428532. doi: 10.3389/fnut.2024.1428532 (PMC11254848; doi:10.3389/fnut.2024.1428532)
Supplement: Supplementary file 2 [file Table_2.DOCX]

| **Supplement Table 2. Performance metrics for six models in the training set.** | | | | | | | |
| --- | --- | --- | --- | --- | --- | --- | --- |
| **Model** | **AUROC (95% CI)** | **Accuracy (95% CI)** | **Sensitivity (95% CI)** | **Specificity (95% CI)** | **PPV (95% CI)** | **NPV (95% CI)** | **F1 score (95% CI)** |
| XGBoost | 0.938 (0.906-0.970) | 0.863(0.857-0.868) | 0.925(0.912-0.938) | 0.847(0.836-0.858) | 0.692(0.678-0.706) | 0.958(0.950-0.965) | 0.791(0.784-0.798) |
| LR | 0.924 (0.887-0.960) | 0.864(0.856-0.872) | 0.921(0.910-0.933) | 0.85(0.839-0.860) | 0.693(0.682-0.705) | 0.959(0.955-0.963) | 0.791(0.781-0.801) |
| RF | 0.963 (0.940-0.986) | 0.899(0.889-0.909) | 0.925(0.906-0.945) | 0.896(0.876-0.916) | 0.77(0.739-0.801) | 0.963(0.956-0.970) | 0.839(0.826-0.852) |
| MLP | 0.949 (0.923-0.975) | 0.871(0.844-0.898) | 0.944(0.920-0.967) | 0.851(0.814-0.887) | 0.707(0.655-0.759) | 0.968(0.959-0.978) | 0.806(0.770-0.842) |
| SVM | 0.906 (0.866-0.946) | 0.821(0.809-0.834) | 0.929(0.911-0.948) | 0.788(0.769-0.808) | 0.619(0.599-0.639) | 0.959(0.951-0.968) | 0.742(0.729-0.756) |
| KNN | 0.872 (0.824-0.920) | 0.769(0.757-0.781) | 0.91(0.886-0.934) | 0.699(0.671-0.728) | 0.552(0.536-0.568) | 0.919(0.906-0.931) | 0.686(0.674-0.699) |
| **XGBoost**, eXtreme Gradient Boost; **LR**, Logistic Regress; **RF**, Random Forest; **MLP**, Multilayer Perceptron; **SVM**, Support Vector Machines; **KNN**, K-Nearest Neighbor; **PPV**, Positive Predicted Value; **NPV**, Negative Predictive Value. | | | | | | | |
