# Supplementary material for: Development of a machine learning-based model to predict major adverse events after surgery for type A aortic dissection complicated by malnutrition
Source: Front Nutr. 2024 Jul 4;11:1428532. doi: 10.3389/fnut.2024.1428532 (PMC11254848; doi:10.3389/fnut.2024.1428532)
Supplement: Supplementary file 3 [file Table_3.DOCX]

| **Supplement Table 3. Performance metrics for six models in the validation set.** | | | | | | | |
| --- | --- | --- | --- | --- | --- | --- | --- |
| **Model** | **AUROC (95% CI)** | **Accuracy (95% CI)** | **Sensitivity (95% CI)** | **Specificity (95% CI)** | **PPV (95% CI)** | **NPV (95% CI)** | **F1 score (95% CI)** |
| XGBoost | 0.857 (0.753-0.961) | 0.776(0.747-0.805) | 0.954(0.921-0.988) | 0.724(0.669-0.778) | 0.559(0.509-0.609) | 0.907(0.865-0.949) | 0.702(0.662-0.742) |
| LR | 0.885 (0.794-0.974) | 0.814(0.787-0.841) | 0.935(0.896-0.974) | 0.771(0.708-0.834) | 0.61(0.565-0.655) | 0.929(0.901-0.957) | 0.734(0.703-0.766) |
| RF | 0.899 (0.815-0.982) | 0.818(0.776-0.860) | 0.969(0.944-0.994) | 0.784(0.731-0.838) | 0.643(0.583-0.703) | 0.909(0.869-0.950) | 0.768(0.729-0.806) |
| MLP | 0.856 (0.756-0.956) | 0.78(0.751-0.809) | 0.938(0.890-0.987) | 0.747(0.696-0.798) | 0.576(0.513-0.638) | 0.905(0.879-0.932) | 0.708(0.655-0.761) |
| SVM | 0.861 (0.755-0.966) | 0.784(0.744-0.824) | 0.907(0.854-0.959) | 0.774(0.713-0.834) | 0.575(0.536-0.614) | 0.925(0.891-0.958) | 0.701(0.666-0.735) |
| KNN | 0.820 (0.701-0.938) | 0.72(0.676-0.764) | 0.894(0.841-0.947) | 0.631(0.545-0.717) | 0.497(0.443-0.551) | 0.879(0.860-0.897) | 0.631(0.592-0.669) |
| **XGBoost**, eXtreme Gradient Boost; **LR**, Logistic Regress; **RF**, Random Forest; **MLP**, Multilayer Perceptron; **SVM**, Support Vector Machines; **KNN**, K-Nearest Neighbor; **PPV**, Positive Predicted Value; **NPV**, Negative Predictive Value. | | | | | | | |
